# Toward the clinical translation of safe intravenous long circulating ILNEs contrast agent for CT imaging

**DOI:** 10.7150/thno.110014

**Published:** 2025-03-21

**Authors:** Mohamed F. Attia, Ryan N. Marasco, Samuel Kwain, Charity Foxx, Daniel C. Whitehead, Alexander Kabanov, Yueh Z. Lee

**Affiliations:** 1 Center for Nanotechnology in Drug Delivery and Division of Pharmacoengineering and Molecular Pharmaceutics, Eshelman School of Pharmacy, University of North Carolina at Chapel Hill, NC 27599, USA.; 2 Department of Chemistry, Clemson University, Clemson, SC, 29634, USA.; 3 School of Medicine, The University of North Carolina at Chapel Hill, Chapel Hill, North Carolina 27599, USA.

**Keywords:** intravenous contrast agents, CT imaging, x-ray imaging, iodinated nanoemulsions, kidney-safe blood pool agents, clinical translation

## Abstract

**Rationale:** X-ray computed tomography (CT) is crucial in precision medicine for diagnostic and therapeutic guidance. However, current small molecule CT contrast agents pose risks such as nephrotoxicity, short blood circulation time, limited scan durations, potential thyroid impact, and immune responses. These challenges necessitate the development of kidney-safe nanoparticle (NP)-based contrast agents (CAs).

**Methods:** We developed safe intravenous blood pool NP-based CT CAs at a clinical-equivalent dose of 300 mgI/kg, suitable for vascular and hepatic imaging. Our iodinated lipid nanoemulsions (ILNEs) were optimized for shelf-life stability, osmolarity, and viscosity for excellent injectability. The ILNEs were designed to offer high contrast and were tested for minimal protein interaction, prolonged blood circulation, and hepatic clearance. *In vitro* studies, along with tests in mice and porcine models, were conducted to confirm safety, cytocompatibility, and absence of tissue damage.

**Results:** The ILNEs demonstrated high x-ray attenuation, improved contrast enhancement, extended stability, and batch-to-batch consistency. They exhibited minimal protein interaction, prolonged blood residency of about 4 h, and hepatic clearance within three days, avoiding nephrotoxicity. Blood and thyroid-stimulating hormone (TSH) analyses, along with kidney and liver function tests, confirmed the safety of ILNEs.

**Conclusion:** Our ILNEs offer a promising alternative to current CT contrast agents, with improved safety and efficacy profiles. The results support further toxicity evaluations for clinical translation, highlighting the potential of ILNEs in vascular and hepatic imaging without the associated risks of nephrotoxicity.

## Introduction

Advancements in medical imaging technologies, particularly Computed Tomography (CT), have revolutionized diagnostic practices by providing high-resolution, three-dimensional images crucial for precisely detecting and monitoring diseases. Central to CT imaging are contrast agents, which enhance tissue and organ visualization and discrimination, especially when identifying solid malignancies with small sizes and at early stages 1, 2. High K-edge energy contrast agents like gold, gadolinium, and bismuth are explored but face safety concerns due to their long-term retention 3-8. Additionally, their synthesis often involves complex and hazardous processes 9, 10. In contrast, iodinated compounds, known for their high x-ray attenuation properties, radiopacity, and biocompatibility, have been essential in clinical practice since 1950, with about 75 million doses administered annually 11. However, traditional iodinated contrast agents have limitations, including rapid clearance, narrow imaging windows, and potential nephrotoxicity, especially in patients with compromised renal function. They can also cause adverse reactions, such as worsened renal impairment and increased risk of post-CT acute kidney injury 12-14, as well as adverse cardiac events within a month post-administration 15, 16.

In that sense, NP-based iodinated contrast agents (ICAs) may enable more targeted imaging and expand diagnostic options, but face hurdles like high excipient amounts, biological and shelf-life stability, large volume doses, production scale-up, and complex chemistry for conjugating high concentrations of iodine. Consequently, none of the developed polymers, micelles, liposomes, and inorganic metal-based nanoparticles have successfully translated into clinical applications 17-22. Innovative contrast agents are needed to overcome these challenges while maintaining diagnostic efficacy and patient safety.

Luo's team has recently synthesized poly(diiododiacetylene) (PIDA), which demonstrates dual visibility in CT imaging and to the naked eye. Their research highlights PIDA's effectiveness in CT-guided preoperative planning, visualization-guided surgery, and imaging-guided radiotherapy, showing comparable efficiency to clinical fiducial markers 23. In another study, they demonstrated that PIDA nanofibers are promising for targeted CT imaging and therapeutic intervention for inflammatory bowel disease (IBD). These nanofibers attach to the gastrointestinal tract's mucus layer, alleviating inflammation while maintaining gut microbiota balance 24. However, PIDA's application is limited to oral and local administration and is unsuitable for intravenous (IV) CT agents due to safety concerns. In parallel, Liu and co-workers have synthesized renoprotective angiographic polymersomes (RAPs) as innovative CT contrast agents to mitigate contrast-induced nephropathy (CIN). These biodegradable nanoparticles, designed with x-ray attenuation and ROS-scavenging capabilities, reduce CIN risk in mice with kidney injury. RAPs also extend angiographic live time by five-fold compared to traditional iodinated agents, offering safer vascular imaging. However, the main drawback is their low iodine content, which requires a high-volume dose, making it insufficient to improve contrast enhancement in humans 25.

Lipid nanoemulsions (LNEs) have emerged as promising candidates for CT contrast enhancement 26, 27. LNEs, characterized by nanoscale droplets of oil or lipid stabilized in an aqueous medium, offer advantages such as improved biocompatibility, tunable physicochemical properties, prolonged circulation times, reduced renal clearance, and potential for targeted delivery. LNEs mimic particles inherent in the body, like intracellular lipid droplets and low-density lipoproteins (LDLs), making them safe platforms for developing imaging agents and nanomedicines 28-30. Incorporating iodinated compounds into LNEs can enhance x-ray attenuation, improving imaging quality while minimizing adverse effects associated with free iodinated molecules. This class of LNEs has shown high integrity *in vivo* and significant passive accumulation in tumor tissues via the enhanced permeability and retention (EPR) effect 31. The key advantage is the facile surface functionalization of LNEs with ligand models or antibodies, facilitating selective targeting of specific tissues with low off-target bindings 32-35.

Translating injectable, safe, radiographic long-circulating ILNE contrast agents from preclinical studies to clinical application represents a significant milestone in molecular imaging. This transition requires thorough characterization of ILNEs' pharmacokinetics, biodistribution, and safety profiles, along with optimizing formulation parameters to ensure efficacy and regulatory compliance.

The focus of this study is to advance the translation of new ILNE contrast agents for clinical CT imaging. By harnessing ILNEs' unique characteristics and incorporating iodinated moieties, we aim to develop contrast agents that enhance imaging quality and exhibit improved safety profiles and pharmacokinetic properties compared to conventional counterparts. Our objectives include evaluating the *in vitro* stability, cytotoxicity, and hemocompatibility of the formulated contrast agents, assessing their *in vivo* pharmacokinetics, biodistribution, and imaging efficacy in preclinical models, and investigating safety and contrast enhancement profiles in relevant clinical settings. By fulfilling these objectives, we seek to bridge the gap between preclinical development and clinical translation of novel intravenous ILNE contrast agents, potentially enhancing diagnostic accuracy and improving patient outcomes in CT imaging.

## Materials and Methods

### Materials

Non-ionic amphiphilic PEGylated surfactants: Kolliphor^®^ HS 15 (Solutol^®^ HS15, a polyethylene glycol (15)-hydroxystearate) and Kolliphor^®^ ELP (Cremophor^®^ EL (CrEL), a polyethoxylated (35) castor oil) were gifts from BASF (New York, USA). Oleic acid, 2,4,6-triiodophenol (TIPh), 4-dimethylaminopyridine (DMAP), *N,N′*-dicyclohexylcarbodiimide (DCC), dichloromethane, ethyl acetate, cyclohexane, sodium hydrogen carbonate (NaHCO_3_), sodium sulfate anhydrous (Na_2_SO_4_), sodium chloride (NaCl), deuterated chloroform (CDCl_3_), were purchased from Sigma-Aldrich (St. Louis, MO). Hoechst 33258 solution, Dil Stain (1,1'-Dioctadecyl-3,3,3',3'-Tetramethylindocarbocyanine Perchlorate ('DiI'; DiIC18(3))) were purchased from ThermoFisher Scientific. Phosphate buffered saline (PBS), human plasma (HP), normal saline (0.9 NaCl), Dulbecco's modified Eagle medium (DMEM), Roswell Park Memorial Institute (RPMI) 1640 Media, penicillin-streptomycin solution (10,000 units penicillin per ml and 10 mg streptomycin per ml), fetal bovine serum (FBS), 0.22 µm syringe filters, and Vybrant™, Cell Counting Kit-8 (CCK-8) were purchased from Thermo Fisher Scientific. Human Dermal Fibroblasts, neonatal (HDFn), RAW 264.7 macrophages and IC21 cells were obtained from the American Type Culture Collection (ATCC). Preclinical Fenestra^TM^ HDVC was purchased from MediLumine Inc. and OptiPrep^TM^ Density Gradient Medium, 60 (w/v) iodixanol in water was purchased from Sigma-Aldrich (St. Louis, MO).

## Methods

### Synthesis of 2,4,6-triiodophenyl oleate (TIPhO)

The synthetic procedures were performed as outlined in our previous works with a product yield of 89% 36. ^1^HNMR analysis was also conducted using the same methods as before 36. The resulting ^1^HNMR data can be found in supplementary **Figure S1**.

TIPhO: Colorless oil; ^1^H NMR (500 MHz, CDCl_3_): δ/ppm: 8.05 (s, 2H), 5.33 - 5.30 (m, 2H), 2.62 (t, *J* = 7.5 Hz, 2H), 2.27 (t, *J* = 7.5 Hz, 1H), 2.04 - 1.94 (m, 4H), 1.82 - 1.76 (m, 2H), 1.59 (t, *J* = 7.3 Hz, 1H), 1.47 - 1.41 (m, 2H), 1.33 - 1.18 (m, 16H), 0.86 (t, *J* = 6.9 Hz, 3H).^ 13^C NMR (126 MHz, CDCl_3_) δ 174.08, 151.86, 146.96, 129.93, 129.69, 91.79, 91.35, 77.37, 77.11, 76.86, 34.05, 31.93, 29.78, 29.69, 29.54, 29.35, 29.33, 29.17, 29.15, 29.13, 29.09, 27.22, 27.16, 24.94, 22.70, 14.12.

### Formulation of ILNEs

The ILNEs were produced using a low-energy spontaneous emulsification technique, as outlined previously 37. The oil phase components, namely TIPhO and Kolliphor ELP surfactant, were typically thoroughly mixed by heating at 50-60 °C and vortexing for 5 min until complete dissolution and miscibility and the emergence of a clear phase. Subsequently, 0.1 NaCl saline (the aqueous phase) was swiftly introduced to the mixture, followed immediately by vortexing for 2-3 min, resulting in the formation of an opaque colloidal nanosuspension of ILNEs. The formulations were designed to maintain a *surfactant / (surfactant + oil)* weight ratio (SOR) at 20-50 wt% and *(surfactant + oil) / (surfactant + oil + water)* weight ratio (SOWR) at 40 wt%. The resultant samples were then passed through a 0.22 µm syringe filter for sterilization and to eliminate any potential aggregates or junctions. These samples were subsequently stored either at room temperature (RT) or at 4 °C. For comparative analysis, some samples underwent lyophilization, followed by rehydration and storage under the same conditions of RT or 4 °C for further characterization.

### NP size and zeta-potential measurements

Dynamic light scattering (DLS) analysis was conducted following the method outlined in previous studies 38. Similarly, nanoparticle tracking analysis (NTA) by Zetaview was performed using a Zetaview PMX 110 V3.0 instrument (Particle Metrix GmbH, Germany), with data analysis conducted using the Zetaview NTA software as previously described 38.

### Cryo-transmission electron microscopy (Cryo-TEM)

Cryo-grids containing ILNE3 samples were swiftly immersed in a 60:40 mixture of liquid ethane and propane using a FEI Vitrobot Mark IV (ThermoFisher Scientific) at 22 °C and 95% humidity. Lacey carbon TEM grids were made hydrophilic via indirect O_2_/Ag plasma treatment with a PIE Scientific TergeoEM plasma cleaner. Each cryo-grid received 3 µL of ILNE3 sample in normal saline buffer on the carbon side of the TEM grid for 30 s, followed by blotting for 3-6 s with Whatman #595 filter paper, and then plunged into the ethane-propane mixture. The cryo-grids were then imaged under low-dose conditions using a 200 keV TFS Talos Arctica equipped with a Gatan K3 DED.

### Ultraviolet-visible (UV-Vis) spectroscopy

Using a Cary 4000 spectrophotometer (Varian), UV-Vis absorption spectra were recorded in the range of 200-700 nm. Absorbance measurements were taken at the peak wavelengths (λ_max_) of 230 nm for CrEL surfactant and 244 nm for TIPhO compounds.

### Viscosity measurements

Particle-tracking microrheology (PTMR) measures the mechanical properties of mucus at the length scale of its constituent biopolymers 39, 40. The procedures were performed according to the reported method 41, 42. Viscosity of ILNE3 and iodixanol was measured using a commercially available viscometer (µVISC, RheoSense). Measurements for all solutions were made at both 23 °C and 37 °C.

### Particle stability in serum and plasma

To assess the stability of NPs in FBS and HP, freshly prepared ILNE3 and ILNE4 in normal saline solution (0.9% NaCl) were separately incubated in FBS and HP at two concentrations (10% and 20% (v/v)). The samples were then placed in shaking incubators set at 200 rpm and 37 °C for over 48 h. Size distribution and polydispersity were monitored using DLS at various time intervals (0, 1, 2, 4, 6, 24, and 48 h).

### Cell viability assessment

We conducted cell viability studies to assess the *in vitro* cytotoxicity of ILNE3 on HDFn, RAW 264.7 macrophages, and IC21 cells. Various concentrations of ILNE3 were prepared through serial dilution in full medium and applied to the cells. Before treatment, cells were cultured and seeded in a 96-well plate at a density of 10^4^ cells per well, allowing them to attach for 24 h. Cell viability was assessed using the CCK-8 assay according to the manufacturer's instructions, 48 h after incubation with ILNE3. The data are presented as the mean ± standard deviation (SD) of six replicate wells.

### Cellular uptake of ILNE3 in RAW264.7 macrophages

A 100,000 RAW 264.7 macrophage cells were seeded per well in an appropriate cell culture plate. A solution of ILNE3 (40 mg NPs/mL) containing 4 µL of 25 mM Dil dye in NP lipid cores was prepared. The 1 mL of ILNE3 solution underwent purification using a nab10 column to eliminate any free dye and excess ingredients, yielding 1.5 mL of purified ILNEs as a stock solution. NTA analysis revealed a concentration of 7.2 x 10^13^ particles/mL, with a ζ-potential of -12.8 mV, and a particle size of 72.9 ± 5.1 nm. Subsequently, two different concentrations of ILNE3, 10 µL and 20 µL aliquots, were separately added to 1 mL of DMEM. This resulted in concentrations of approximately 266 µg NPs/mL, Dil dye of ~130 nM, and 7.2 x 10^11^ particles/mL for the lower concentration, and approximately 533 µg NPs/mL, Dil dye of 260 nM, and 14.4 x 10^11^ particles/mL for the higher concentration. The 100,000 cells were then incubated with these two respective ILNE3 concentrations for 4 and 24 h. Following the incubation period, the media was removed, and the cells were washed twice with fresh warm DMEM (1 mL). Subsequently, 0.5 mL of Hoechst dye solution (2 µg/mL) was incubated with the cells for 20 min. The dye solution was then removed, and the cells were subjected to two additional washes with DMEM. Finally, 0.5 mL of DMEM was added to each well, and the cells were imaged using a fluorescence microscope.

### Phantom scan: assessment of x-ray attenuation for quantification of iodine concentration

The samples were scanned using a Rigaku Quantum GX small-animal CT system. Imaging parameters included an x-ray voltage of 90 kVp, an anode current of 88 μA, and an exposure time of 120 ms per rotational step (total rotation: 360 degrees, 180 steps). Reconstructed images were generated on a 512 × 512 pixel grid with a pixel size of 66.7 μm × 66.7 μm. ILNE contrast agents were scanned alongside various concentrations of the clinical CT contrast agent Visipaque™ (iodixanol), with deionized water serving as the control (0 HU).

### Animal studies

All animal procedures adhered to the guidelines set by the Institutional Animal Care and Use Committee (IACUC) at the University of North Carolina at Chapel Hill. Healthy male C57BL/6 mice, aged 6-8 weeks, were sourced from Jackson Laboratory. The mice (five per cage) were randomly assigned to control and contrast agent groups. Animals were anesthetized and intubated before any procedure with the assistance of the Division of Comparative Medicine (DCM). All non-imaging procedures were performed by DCM staff.

Sequential imaging was performed on the Quantum GX2 micro-CT scanner which allows rapid, repeated scans of the same region. A standard abdominal imaging protocol was utilized with isoflurane anesthesia. We used this approach to replicate the four-phase liver CT imaging approach on clinical scanners. Images were acquired during the administration of the ILNE contrast agent at total iodine doses of ~300 mgI/kg. Dynamic images were acquired every 5 s for 60 s during which a tail vein injection of the contrast agent will be administered. Repeat single-phase scans were subsequently at 5 min, 1, 2-, 4-, 24- and 72 h post-injection. For this study, healthy mice were utilized. A reference sample of the contrast agent was scanned within the field-of-view of the liver to provide an external reference for the degree of iodine attenuation.

Analysis: Regions of interest (ROIs) were drawn on the kidney, spleen, liver, aorta, and heart to evaluate the attenuation of the injected contrast agent in Hounsfield Units (HU). We evaluated the relative hepatic uptake of the contrast agent as measured by increased attenuation.

The dose-escalation toxicity study involved the administration of a single dose of 0.9% saline solution and freshly prepared contrast agent formulations at concentrations of 300 mgI/mL (equivalent to 800 mg ILNEs/mL) and 750 mgI/mL (equivalent to 2000 mg ILNEs/mL) in 0.9% saline solution via tail vein intravenous (i.v.) injection. The general behavior and changes in body weight of the mice were monitored and recorded every other day for up to 10 days.

### Hematology and blood chemistry studies

Blood samples were collected from C57BL/6 mice at three and ten days post-ILNE3 injection. Using 25 G syringes, blood was drawn via cardiac puncture into K2-EDTA microtainer tubes (500 µL; BD 365974, USA). Cell blood count and hematology parameters were measured with an IDEXX Procyte DX (Westbrook, Maine, USA). Plasma was separated by centrifuging the blood samples at 2,000 x G for 15 min, and blood chemistry parameters were analyzed using an Alfa Wassermann Vet Axcel (West Caldwell, NJ, USA) blood chemistry analyzer.

### Histopathological assessment

Three days post-ILNE3 injection, tissues (liver, spleen, kidney, heart, and lung) were collected and preserved in 10% neutral buffered formalin (Sigma-Aldrich) for histological examination. The histological analysis was conducted by the Center for Gastrointestinal Biology and Disease Core at UNC, following previously described methods 43.

### Porcine animal model

Imaging of a 16.6 kg porcine was performed on a clinical CT scanner (Siemens Force) using an abdominal protocol at 120kVp and mean x-ray tube current of 32mA. Images were reconstructed with a standard soft tissue kernel. All studies were performed under IACUC approval and with the assistance of DCM veterinarians. Imaging was acquired at 2 h after the i.v. administration of the ILNE3 contrast agent (300 mgI/mL). Iohexol (300 mgI/mL) based imaging was performed in a different animal for reference prior to and after the i.v. administration of contrast agent at 1.5 mL/kg. Imaging was performed during the arterial phase of contrast administration. Vital signs were continuously monitored per protocol. No acute changes in the animal's vital signs were observed during the administration of contrast or during the scan.

## Results

### Preparation and characterization of ILNE CT contrast agents

**Figure 1:**
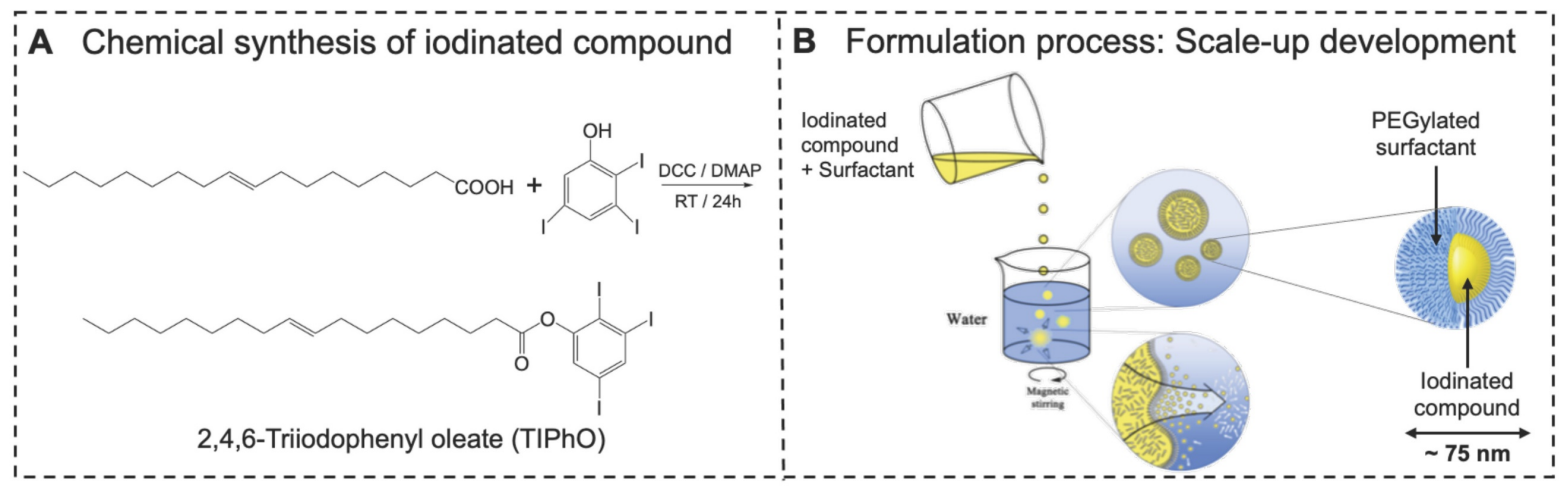
**Preparation of ILNEs CT contrast agent.** (**A**) Chemical synthesis of TIPhO via a one-step esterification reaction. (**B**) The formulation of ILNEs by a low-energy spontaneous emulsification process produced stable nanoemulsion droplets in the 30-150 nm size range, depending on the SOR ratio. Higher concentrations of the iodinated compound resulted in larger droplet sizes.

We synthesized an iodinated lipid compound, TIPhO, achieving an 89% yield through a one-step esterification reaction using EDC•HCl and DMAP (**Figure 1A**). The chemical structure was confirmed via ^1^HNMR and ^13^CNMR (Data are represented in the methods section and the charts in supplementary **Fig S1**), revealing corresponding signals assigned to the molecule, consistent with our previous reports 36.

Using a green and scalable process, we employed a low-energy spontaneous emulsification technique without organic solvents or high-energy inputs such as high-pressure homogenization or high-temperature methods. The production of LNEs commonly necessitates significant energy deposition and strong shear forces to overcome interfacial tension during droplet formation, while spontaneous emulsification approach offers an alternative that does not require high pressure or energy. This method not only saves time and money but also reduces the risk of material quality degradation. It also facilitates scalable production from research and development to pilot scale to production scale with consistent, linearly scalable results. It brings several advantages, including efficient size reduction with a uniform size distribution, essential for producing stable nanoemulsions with enhanced bioavailability and longer shelf-life. It enables sterile filtration with minimal clogging and the production of transparent emulsions.

Our approach involved mixing the two ingredients-iodinated compound (TIPhO) and nonionic amphiphilic PEGylated surfactant Kolliphor^®^ ELP to form the oil phase at SOR of 20-50 wt%. The aqueous phase (0.9% normal saline) was then added at a SOWR of 40 wt% (**Fig 1b**). This produced opaque, uniform, and homogeneous formulations that remained stable for months and even extended to years at RT or 4 °C. It is worth noting that the surfactant played a crucial role in reducing the interfacial tension at the oil/water interface and preventing nanodroplet aggregation, as well as the low-density oil phase, altogether forming an exceptionally stable nanoformulation.

**Figure 2:**
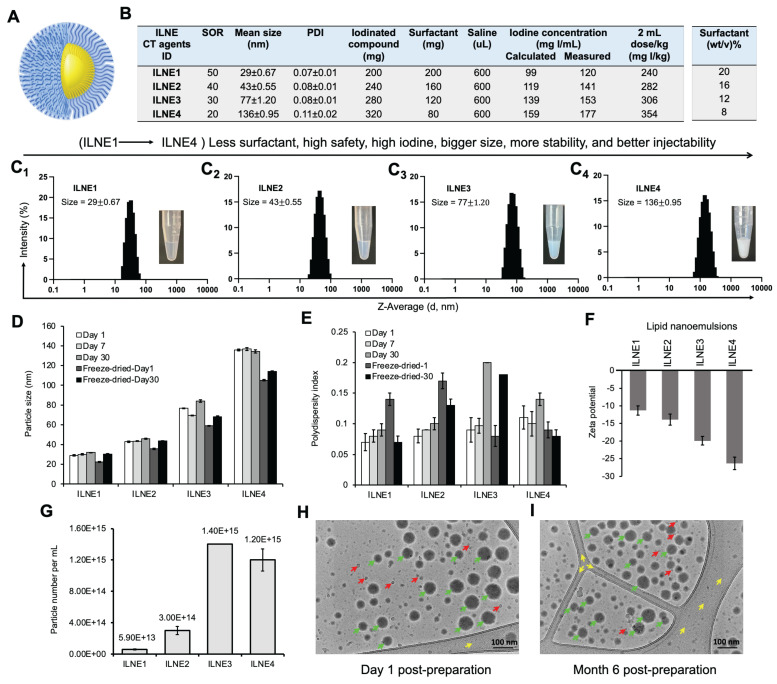
**Characterization of the formulated lipid nanoemulsions.** (**A**) Schematic representation of ILNEs composed of an oily core (yellow) surrounded by a PEG shell (blue). (**B**) Table showing the composition and ratio of all ingredients involved in the ILNE formulations (ILNE1-4) at SOWR 40%, and the final iodine concentrations of the injected dose. (**C_1_-C_4_**) Appearance and DLS histograms showing the size distribution of the four ILNEs. (**D**,**E**) Particle size and PDI over time measured by DLS. Samples were kept in aqueous dispersion at RT for different time intervals. Freeze-dried-1 and freeze-dried-30 are the redispersed lyophilized samples after one and 30 days of preparation, respectively. (**F**) Particle ζ-potential measured by Malvern Zetasizer. (**G**) Particle concentration of ILNEs determined by NTA (n = 3). (**H**,**I**) Cryo-TEM images of ILNE3 at one day (h) and six months (i) post-preparation at RT. The green arrow indicates ILNE3 particles, the red arrow indicates ice particles, and the yellow arrow indicates the grid membrane.

Four ILNE formulations (ILNE1-4) with iodinated core and surface PEG molecules (**Figure 2A**) were designed with varying SOR ratios, affecting hydrodynamic size, ζ-potential, viscosity, and iodine content (**Figure 2B**). SOR30 ILNEs were optimized for injectability, viscosity, and achieving a clinical dose relevant to clinical CT contrast agents. Our goal was to minimize excipients and maximize iodinated molecules to mitigate cytotoxicity and increase contrast enhancement thus lowering the volume dose injected in patients to a clinically relevant dose of about 300 mg/kg.

Our previous efforts to formulate efficient nanodroplet emulsions of lipidic compounds with free active functional groups (COOH or NH_2_) encountered challenges in achieving the desired physicochemical properties, injectability, and bioavailability 34, 36. These difficulties often arise from the physical characteristics and/or chemical interactions, such as hydrogen bonding, Van der Waals forces, and ionic forces, between these functional groups and surfactants. Blocking these groups was found to improve homogeneity with surfactants, enhancing formulation efficiency. We observed that unmodified oleic acid formed highly viscous nanogels at lower concentrations (SOR 15-20 wt%) and solidified at higher concentrations (SOR > 20 wt%), resulting in less efficient nanoformulations. LNE1-4 of noniodinated lipid (oleic acid itself), yielded less efficient suspension with relatively large particle sizes (~300-500 nm) and PDIs (> 0.4) (**Figure S2**). In contrast, as determined by DLS, ILNE1-4 produced narrowly distributed sizes (30-135 nm) and low PDIs (<0.1), depending on the SOR ratio (**Figure 2C_1_-C_4_**). Sizes remained stable after 30 days, with a slight size reduction in lyophilized samples (**Figure 2D**). Polydispersity was consistently <0.2, indicating high uniformity and stability **(Figure 2E)**. Sizes and PDIs reduced after freeze-drying (**Figure S3**). Surface charge, determined by DLS Malvern Zetasizer, showed elevated negative ζ-potential with increasing TIPhO (lipid core) concentrations, ranging from -11.3 ± -1.32 to -26.3 ± -1.78 mV (**Figure 2F**).

Particle concentrations of all ILNE contrast agents, determined by Zetaview NTA analysis, revealed distinct particle numbers based on hydrodynamic size and SOR ratio in the suspension: ILNE3 > ILNE4 > ILNE2 > ILNE1 with concentrations of 1.40E+15, 1.20E+15, 3.00E+14, and 5.9E+13 particles/mL, respectively (**Figure 2G**). ILNE3 was selected as the ideal contrast agent for all subsequent studies based on stability, viscosity, contrast efficiency, and clinical dose. Further, Cryo-TEM images of ILNE3 showed similar morphology, size, and homogeneity for fresh and 6-month post-preparation samples as indicated by green arrow (**Figure 2H-I**), consistent with DLS data. The particles exhibited a spherical shape with a black dense color, possibly due to the iodine contrast agent.

The potential translation of contrast agents hinges on various critical factors, including batch reproducibility, scale-up production, and long-term shelf stability and storage, all while prioritizing safety and maximizing contrast. In light of these requirements, we assessed ILNE3 across different batch scales (2 mL, n = 7 separate formulations; 35 mL, n = 1), revealing exceptional consistency across batches (**Figure S4**). Particle sizes centered around 76.6 ± 1.2 nm with a low PDI of 0.08 ± 0.01. Shelf-life stability of ILNE3 and ILNE4 over a year at room temperature showed no significant size alterations, maintaining sizes of 81.1 ± 1.1 and 137 ± 3.8 nm, respectively, with PDIs < 0.2 (**Figure S5**). ILNE3 size and PDI also remained stable over six months at 4 °C (**Figure S6**).

In comparison, we evaluated the characteristics of a commercial preclinical blood pool CT agent, Fenestra^TM^ HDVC, revealing a larger size of 98 ± 0.62 nm and a relatively high PDI of 0.37 ± 0.01, associated with aggregates and increased viscosity. In contrast, our ILNE3 formulation stands out for its clarity, homogeneity, and injectability, coupled with a higher iodine payload (**Figure S7**).

In this context, we assessed the potential of solutol^®^ HS15 (PEG surfactant) as a replacement for CrEL in ILNE formulations, achieving similar iodine concentrations (~353 mgI/kg) as in ILNE4. However, all formulations exhibited larger particle sizes as compared to CrEL-based formulations (**Table S1a**). We also evaluated mixed surfactants; CrEL:solutol (50:50), and successfully developed efficient ILNEs with sizes comparable to Cremophor-based formulations (**Table S1b**). Injected volume doses of ILNE3 across different body weights are presented in **Table S2**.

**Figure 3:**
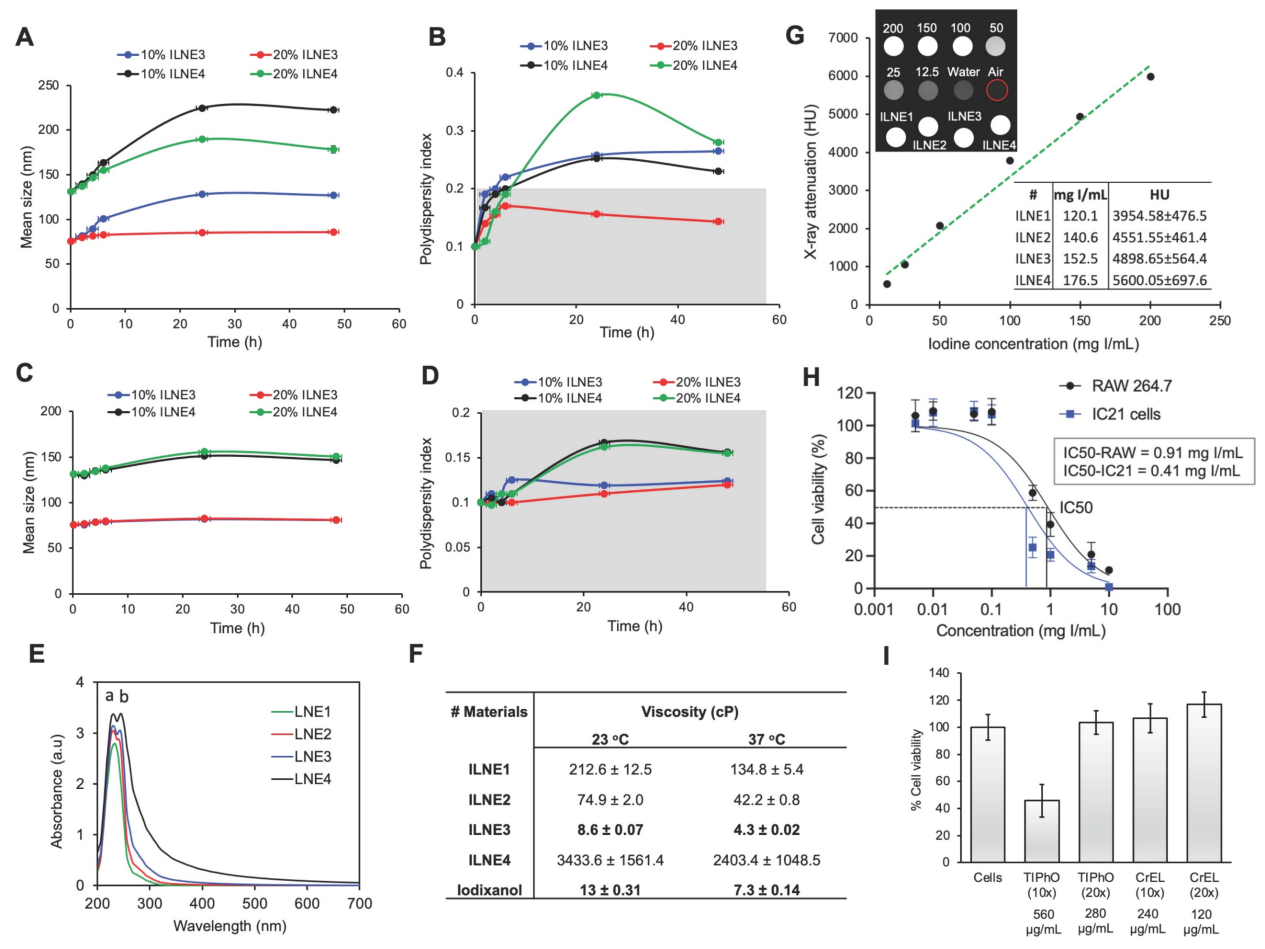
**Characterization and stability of ILNEs in various conditions.** (**A-B**) Particle size stability in HP and (**C-D**) FBS: 10% and 20% (v/v) ILNEs were incubated with (**A-B**) HP or FBS (**C-D**) for 48 h. Particle size (**A-C**) and PDI (**B-D**) were measured at different time points using DLS. **(E)** UV-vis absorbance measurements of ILNE1-4, showing the peaks of CrEL and TIPhO at λmax of ~230 nm and ~244 nm, respectively. **(F)** Viscosity measurements of ILNE1-4, compared with iodixanol stock solution (300 mgI/mL) at 60% (wt/v). **(G)** Phantom scan of ILNEs: *In vitro* evaluation of x-ray attenuation properties (in HU) of ILNE1-4 using a calibration curve made by various dilutions of the iodixanol injection as a reference (expressed in mgI/mL). Air and water were used for normalization. Top view of Eppendorf tubes and source data are provided as a Source Data file. **(H)** CCK-8 cytotoxicity assay of immune cells: Macrophages RAW264.7 and IC21 cells were incubated with ILNE3 for 48 h. Data are presented as the average ± SD (n = 6). **(I)** Cell viability studies of ILNE components: Two concentrations of CrEL and TIPhO were incubated with RAW 264.7 cells for 24 h.

Nanoparticles are often prone to degradation, either *in vitro* or *in vivo*, due to several reasons such as chemical instability, pH sensitivity, aggregation, and biological degradation through interaction with biomacromolecules, rendering them ineffective for delivering therapeutics or contrast agents. Therefore, we assessed the particle stability/degradation for both ILNE3 and ILNE4 following their incubation in both human plasma (HP) and fetal bovine serum (FBS) over a period of 48 h using DLS analysis. Two concentrations, 10% and 20% (v/v) of ILNEs in HP and FBS, were placed in an orbital shaking incubator set at 200 rpm and 37 °C. The particle size and PDI were monitored over time (**Figure 3A-D**). Results showed consistent sizes and PDIs in FBS, with a slight size increase in HP at 10% (v/v) after 24 h, indicating good stability in both environments. This indicates a lack of significant nano-bio interaction and minimal protein binding, thus reflecting considerable stability.

To further characterize the resulting ILNEs, UV-vis absorbance analysis was conducted on ILNE1-4 at a 3200x dilution of the stock solution. This analysis revealed two distinct peaks of maximum wavelengths (λ_max_) at approximately 230 nm (a) and 244 nm (b), assigned to the CrEL surfactant and TIPhO compounds, respectively, confirming the composition of the ILNEs (**Figure 3E**). Additionally, absorbance spectra of all ILNEs were monitored at various dilutions (1600x, 3200x, 6400x, and 12800x), yielding consistent data (supplementary **Figure S8**).

Viscosity is important for designing injectable products. Thus, we evaluated the viscosity of all ILNE formulations and compared them with the clinical CT contrast agent iodixanol (Visipaque™) (300 mgI/mL) at 60% (wt/v). ILNE3 demonstrated lower viscosity than other ILNEs, measuring 8.6 ± 0.07 and 4.3 ± 0.02 cP at 23 °C and 37 °C, respectively. These values were also lower than those of iodixanol, which measured 13 ± 0.31 cP at 23 °C and 7.3 ± 0.14 cP at 37 °C (**Figure 3F**). ILNE3 also had lower viscosity than clinical iohexol (300 mgI/mL) and preclinical Fenestra™ HDVC products.

Iodine content is crucial for CT contrast agents. Most current clinically available hydrophilic CT contrast agents have iodine contents below 50%, such as iodixanol (49.1 wt%), iohexol (46.4 wt%), and iopromide (48.1 wt%). Our ILNEs, with 51.7 wt% iodine, show excellent x-ray attenuation. The CT value, measured in Hounsfield units (HU), increased linearly with iodine concentration in ILNE suspensions (**Figure S9**), which suggests that ILNE3 was well dispersed in the medium. We quantified iodine concentration of all ILNEs using x-ray imaging and a calibration curve with iodixanol dilutions as a reference (**Figure 3G**). Data showed slightly higher x-ray attenuation for ILNEs than theoretical values (**Figure 2B**). Depending on SOR ratios, the iodine concentration ranged from approximately 120 to 176 mgI/mL, affording around 3955 to 5600 HU. Lower SOR values resulted in higher iodine content and contrast. ILNE3 at SOR30 and ILNE4 at SOR20 are promising for clinical CT imaging, allowing low-volume dosages (~1.5-2 mL/kg) with iodine amounts aligning with clinical doses (~300 mgI/mL). ILNE tubes showed similar contrast enhancement to 200 mgI/mL iodixanol, ensuring high contrast (**Figure 3G**).

Next, we assessed the *in vitro* toxicity of ILNE3 suspensions using the CCK-8 test after a 48 h incubation with RAW 264.7 macrophages and IC21 immune cells at various concentrations (**Figure 3H**). The data revealed lower cytotoxicity for RAW macrophages compared to IC21 immune cells, with LC50 values of 0.91 and 0.41 mgI/mL, respectively. Given that ILNE3 suspensions are administered at 40 µL per mouse (20g), containing nearly 2 mL of blood, the injected dose amounts to 8 mg NPs/mL of mouse blood (equivalent to ~2.9 mgI/mL mouse blood), as iodine constitutes approximately 36% of the final NPs. In cytotoxicity studies, the calculated concentration of NPs for *in vitro* cell incubation is considered to be 100-fold less than that administered in animals. As shown in **Figure 3H**, concentrations up to 0.1 mgI/mL demonstrated 100% cell viability in both immune cell types, representing a 29-fold lower concentration than the injected dose in mice, indicating excellent biocompatibility. Moreover, the IC50 values for RAW 264.7 and IC21 cells were 11- and 5-fold less than the administered dose in mice, respectively. These results are notable, considering the CCK-8 assay conditions are more stringent than *in vivo*. During the 48 h incubation *in vitro*, cells are in prolonged contact with ILNEs and free surfactants, whereas these surfactants are generally rapidly cleared from the bloodstream *in vivo*.

We also conducted separate cell viability studies on the CrEL and TIPhO compounds, which constitute ILNE3 (**Figure 3I**). Following a 24 h incubation with RAW macrophages, full cell viability was maintained at concentrations of 240 and 280 µg/mL for CrEL and TIPhO, respectively—these concentrations represent a 20-fold reduction of the injected dose in mice. Upon increasing both concentrations 10-fold, CrEL did not adversely affect the cells, while TIPhO led to approximately 50% cell death among macrophages. Collectively, ILNE3 and its constituents demonstrated exceptional cytocompatibility *in vitro* relative to the injected dose in animals. To further confirm cytocompatibility, we ran cytotoxicity tests on human fibroblast (HDFn) cells treated with various dilutions of ILNE3 and the clinical agent iodixanol for 24 h (**Figure S10**). The results showed that both contrast agents exhibited 100% cell viability up to 500 µg/mL, with viability declining to about 65% for ILNEs at 1000 µg/mL. This demonstrates the safety profile of both materials, although iodixanol appears to be slightly safer.

### *In vitro* cellular uptake study

**Figure 4:**
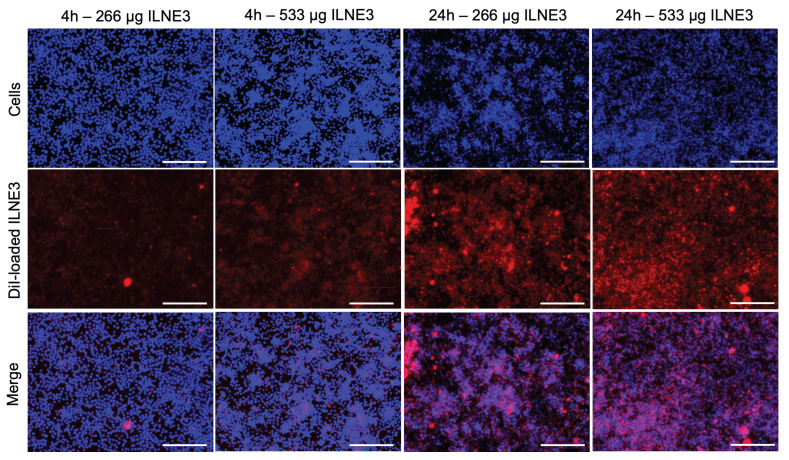
**Cellular uptake study of Dil dye-loaded ILNE3 in RAW 264.7 macrophage cells.** Two concentrations of ILNE3 were incubated in the cells, and the internalization of particles was observed using an optical fluorescence microscope at 4 and 24 h time points. Images in each column (top to down) show: Hoechst dye (blue) for staining cell nucleus, Dil dye-labeled ILNE3 (red), and overlapping (blue-red). The scale bar indicates 150 µm.

Understanding nanoparticle uptake by cells is crucial for evaluating toxicity and biocompatibility. Inadequate internalization or cytotoxic effects can compromise cellular function. NPs must overcome biological barriers like the cell membrane and endosomal/lysosomal compartments to deliver therapeutic or contrast agents effectively. We prepared two concentrations of ILNE3 (266 and 533 µg/mL), encapsulating Dil lipophilic dye within their lipidic core, to monitor their internalization into RAW 264.7 macrophages (**Figure 4**). These samples were incubated with the cells for 4 and 24 h. After incubation, the cells were washed, and the nuclei were stained with Hoechst dye before imaging. The images (**Figure 4**) showed that the higher concentration of particles exhibited slightly greater uptake after 4 h. However, at both concentrations, ILNE3 displayed similar uptake intensity at 24 h, significantly higher than at 4 h. Overall, ILNE3, with a size of about 72.9 ± 5.1 nm and a ζ-potential of -12.8 mV (measured by NTA-Zetaview), demonstrated remarkable internalization within the cytoplasm of macrophage cells, indicating their potential for targeting and biocompatibility.

### *In vivo* CT contrast and pharmacokinetics in mice

**Figure 5:**
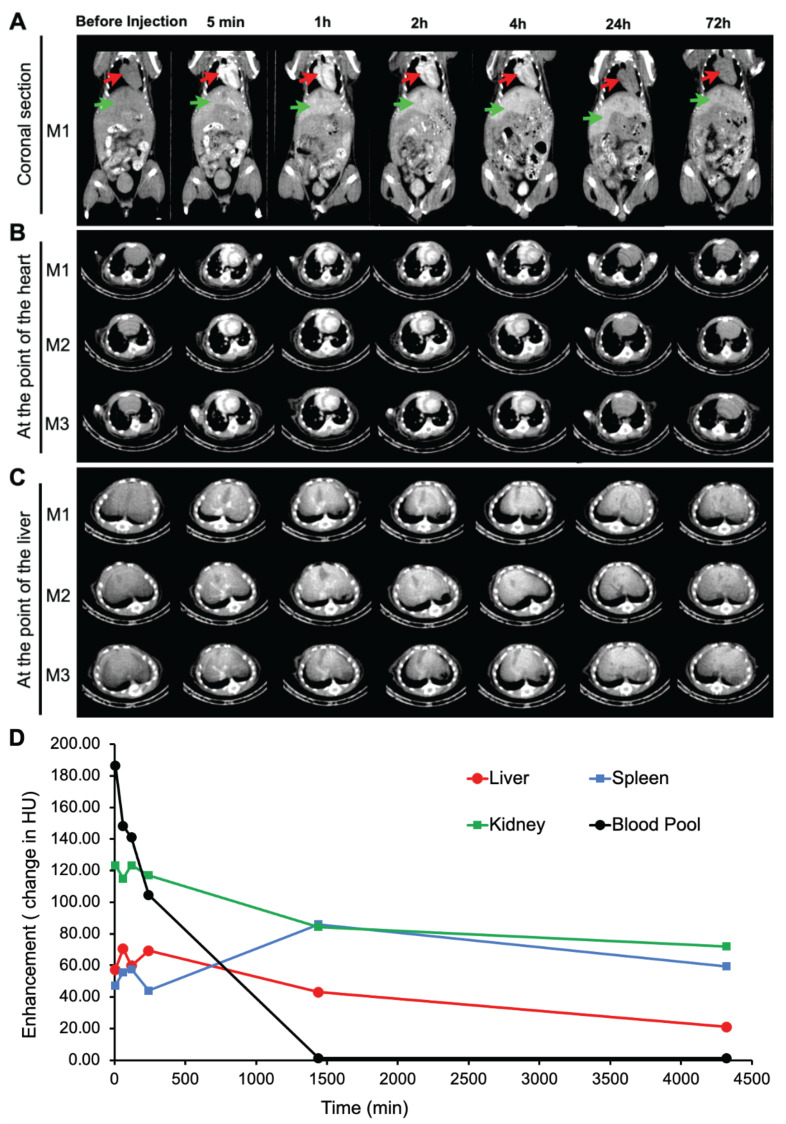
***In vivo* micro-CT imaging, longitudinal studies of biodistribution after i.v. administration of ILNE3 in C57BL/6 mice.** Mice IDs are M1-M3 (n = 3, biologically independent samples). Coronal and axial slices at representative times: (**A**) coronal view of mice pre- and post-injection at different time points. The red arrow represents the heart, and the green arrow represents the liver. (**B**) Transverse section at the view of the heart. (**C**) Transverse section at the view of the liver. (**D**) Quantitative analysis of the x-ray attenuation values in HUs where the ROIs were placed in the heart, liver, spleen, and kidneys.

Inspired by the remarkable x-ray attenuation ability of our ILNEs, we administered ILNE3 intravenously at 2 mL/kg to C57BL/6 mice. Enhanced contrast was observed in the heart and liver compared to pre-injection images, evident in both coronal and transverse views (**Figure 5A-C**). This confirms the rapid distribution of ILNE3 throughout the vasculature, highlighting the major veins of the heart ventricles, aorta, and liver. Over 72 h, no clinical signs of disorder or toxicity were observed in mice. ILNE3 consistently exhibited enhanced contrast, distribution, and clearance behavior. The images revealed enhanced contrast in the heart and a gradual increase in liver contrast up to 4 h post-injection. By 24 h, ILNE3 had been entirely cleared from the bloodstream, with no detectable signals, while notably accumulating in the liver, resulting in pronounced contrast. By 72 h, CT signals in the liver were significantly reduced, confirming elimination from the body. Collectively, ILNE3 functions as a blood pool CT contrast agent (BPCA), boasting a half-life of at least 4 h, prolonging the scan window, and subsequently undergoing hepatic metabolism, circumventing renal clearance. It is effectively eliminated from the body within approximately 3 days post-injection. These attributes match the criteria for optimal BPCAs in clinical settings, featuring a kidney-safe formulation with improved contrast enhancement, followed by rapid clearance from the body post-scanning.

Contrast enhancement was measured in the blood pool (left ventricle ROI), liver, spleen, and kidneys over three days (**Figure 5D**). Blood pool enhancement peaks at the time of injection, achieving nearly 200 HU relative to the background. Although the aorta should match the blood pool, its small size makes it difficult to measure. The kidney parenchyma shows expected vascular enhancement, with no significant contrast accumulation in the collecting system despite parenchymal enhancement. Liver enhancement peaks hours after injection as the agent accumulates in the liver. There is some renal excretion of iodine due to the ultrasmall particles within the ILNE3, with the bladder showing peak enhancement around the 4 h mark based on mouse images (**Figure 5A-C**).

**Figure 6:**
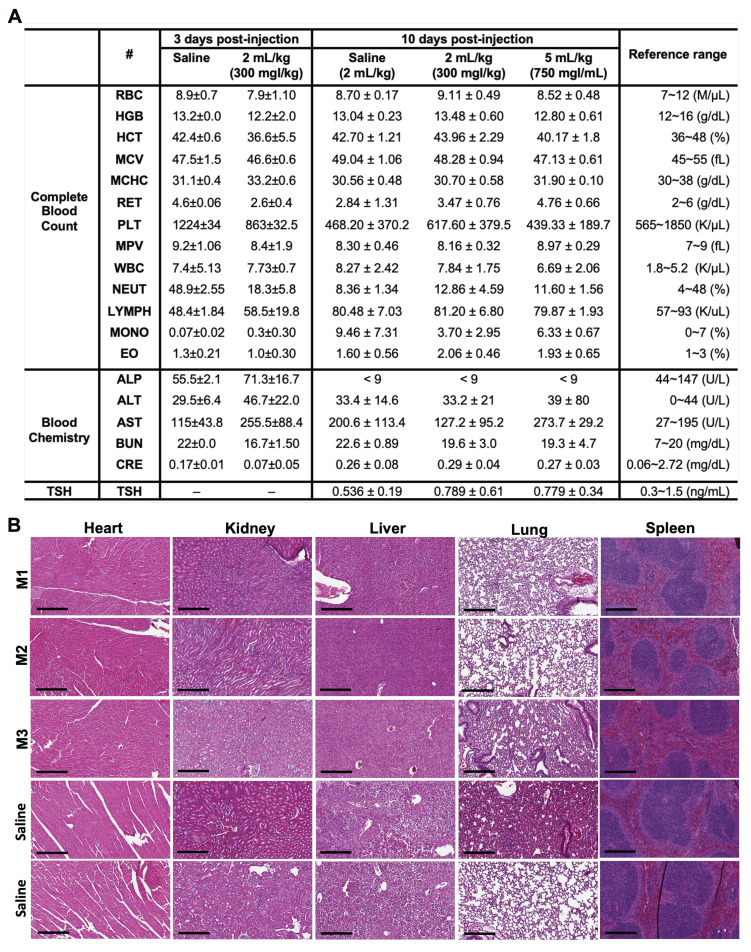
** Blood analysis and histological examination post-ILNE3 injection.** (**A**) Blood analysis and liver/kidney function tests were conducted at two distinct time points: three days following the injection of saline (n = 3 biologically independent mice) and ILNE3 (2 mL/kg, n = 3 biologically independent mice), and ten days post-injection of saline (2 mL/kg, n = 5), ILNE3 (2 mL/kg, n = 5), and ILNE3 (5 mL/kg, n = 3). Thyroid-stimulating hormone (TSH) analysis was also determined at 10 days post-injection. Data are presented as Mean ± SD. Abbreviations: RBC (red blood cells), HGB (hemoglobin), HCT (hematocrit), MCV (mean corpuscular volume), MCHC (mean corpuscular hemoglobin concentration), RET (reticulocytes), PLT (platelets), MPV (mean platelet volume), WBC (white blood cells), NEUT (neutrophils), LYMPH (lymphocytes), MONO (monocytes), EO (eosinophils), ALP (alkaline phosphatase), ALT (alanine aminotransferase), AST (aspartate aminotransferase), BUN (blood urea nitrogen), CERA (creatinine). (**B**) H&E staining histology of organs (heart, kidney, liver, lung, and spleen) harvested three days post-injection of ILNE3 at 2 mL/kg (equivalent to 800 mg ILNE3/kg, n = 3) compared with saline-injected mice (n = 2). (M1-M3) are mice IDs. Scale bar is 300 µm.

To further ensure the safety profile of ILNE3 for prospective applications as CT contrast agent, we performed two experimental designs on C57BL/6 mice, varying in time points and escalating doses: Experiment (I), we compared a control group (n = 2) received saline to an ILNE3-injected group (n = 3) at a dose of 300 mgI/kg. Three days post-injection and CT scanning, both groups were sacrificed for blood analysis and histological examination of major organs (heart, liver, kidney, lung, and spleen). Analysis showed no significant differences in liver/kidney functions or complete blood count (CBC) between the groups (**Figure 6A**). Macroscopic examination and H&E stains indicated no visible damage, toxicity, necrosis, or pathological alterations in tissue architectures (**Figure 6B**). These findings, showcasing normal histological features with no deviations, provide compelling evidence of the nontoxic nature of ILNE3. Experiment (II), three groups were compared: saline-injected mice (n = 5), ILNE3-injected mice at 300 mgI/kg (n = 5), and ILNE3-injected mice at 750 mgI/kg (n = 3). After 10 days, CBC, blood chemistry, and TSH levels were analyzed. Data showed no significant weight changes in mice (**Figure S11**) or differences in blood analysis and liver/kidney function tests (**Figure 6A**). AST levels slightly elevated three days post-injection but normalized after 10 days. The higher dose of 750 mgI/kg (a 2.5-fold increase of clinical dose) showed only a minor AST increase, suggesting ILNEs accumulate in the liver before dissipating. These findings support ILNE3's exemplary biosafety for translational studies.

### *In vivo* CT contrast in porcine model

**Figure 7:**
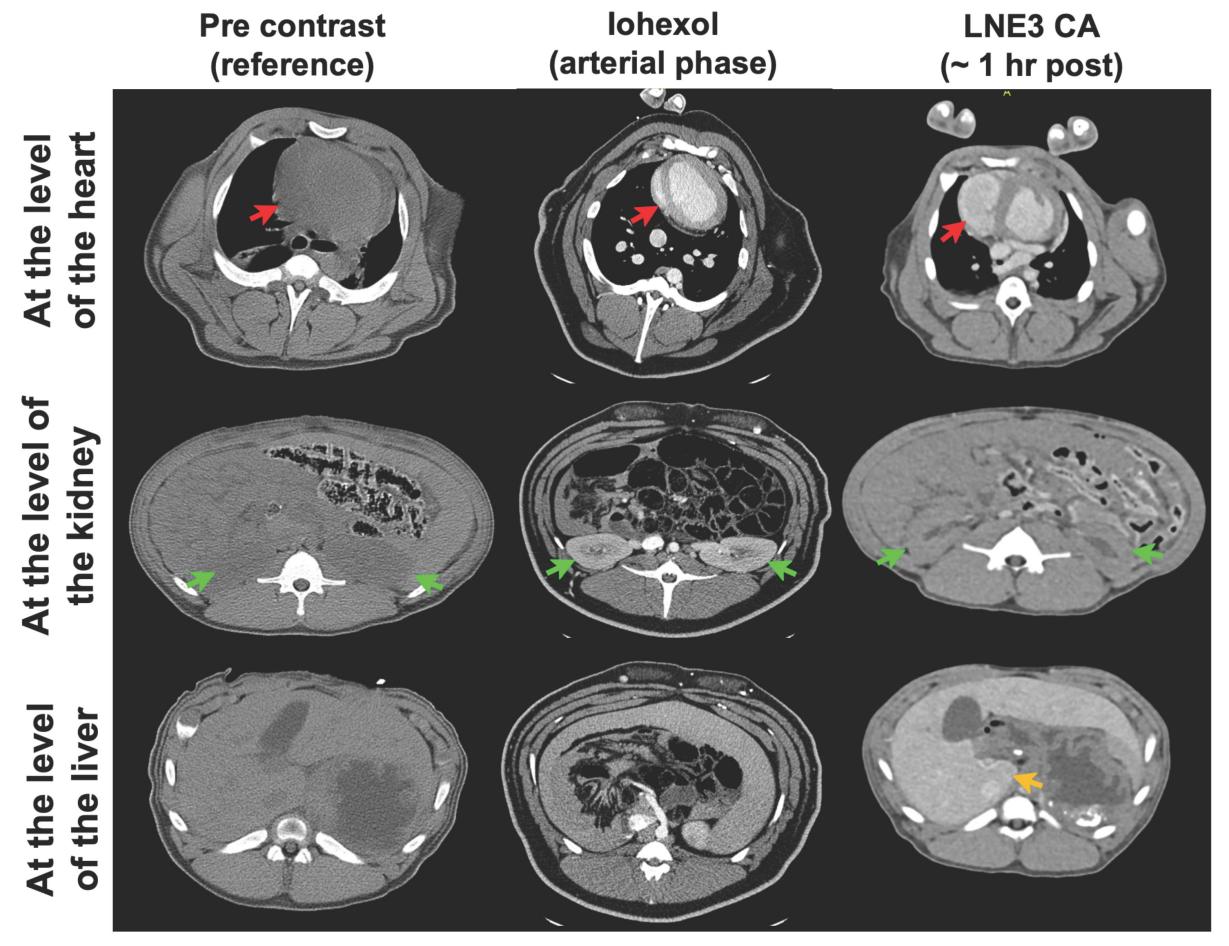
***In vivo* CT imaging of a porcine animal model injected with ILNE3 at a dose of 300 mgI/mL compared to a similar dose of iohexol. Imaging of the iohexol was acquired during the arterial phase, at 30 s post-injection.** ILNE3 imaging was performed at approximately 1 h post injection due to our workflow. Notably, the heart, kidney, and liver were identified and indicated by red, green, and yellow arrows, respectively, facilitating clear visualization and comparison of contrast enhancement dynamics between the two contrast agents. Significant arterial enhancement remains at the 1 h time point with the ILNE3 imaging, despite the significant delay in imaging. Increased liver enhancement is also seen, due to the liver uptake.

To further verify scalability and safety, we injected ILNE3 intravenously into a 16.6 kg porcine at 300 mg/kg (~2 mL/kg) and compared it to clinical iohexol at a similar dose, using the pre-contrast state as a baseline (**Figure 7,** left column). For iohexol, we scanned the porcine at 30 s (arterial phase) due to rapid renal clearance. Improved contrast was observed in the heart (**Figure 7,** middle column), followed by significant kidney contrast. In contrast, ILNE3-injected porcine scanned 1 h post-injection (**Figure 7**, right column) showed heart visualization and enhanced liver contrast with minimal kidney imaging. This suggests ILNE3's potential as a kidney-safe BPCA.

## Discussion

Nano-CT contrast agents are primarily used in preclinical studies involving polymers 23, 44, liposomes 19, 20, and metallic materials 17. Our approach focuses on engineering safe, scalable intravenous iodinated lipid nanodroplet emulsion (ILNE) CT contrast agents. These agents offer significant contrast and minimal toxicity tailored for clinical use, addressing the shortcomings of current clinical and NP-based agents due to their excellent biocompatibility, high iodine payloads, and robust CT intensity signals. Additionally, their surface can be easily labeled with targeted antibodies, enhancing CT imaging specificity and sensitivity. Our ~75 nm-sized ILNEs enable extended blood circulation, expanding the scan window and allowing slower injection rates, minimizing infusion reactions and avoiding renal excretion, thus reducing kidney injury risks.

Previous iodinated contrast agents developed by our team and others suffer from long-term stability issues and excessive surfactant content (50-70 wt%) 37, 45. This impedes clinical development due to toxicity from excessive excipients and the need for higher doses for effective contrast. These formulations also show prolonged accumulation in animals, increasing toxicity risks 37, 45. The chemical structure and composition ratio between surfactant and iodinated compounds in nanoformulations significantly affect size, surface charge, iodine content, blood circulation time, pharmacokinetics, and biodistribution 36, 37, 45. Hydrophilic small molecules with ultrasmall size are rapidly taken up by the kidneys, potentially causing kidney dysfunction, akin to clinical CT agents. Conversely, nanosized formulations (20-200 nm) undergo hepatic pathways and preferentially accumulate in tumor vasculatures due to the enhanced permeability and retention (EPR) effect, aligning with our contrast agents.

In our approach, we employed a mix-and-match strategy to screen various molecules, placing particular emphasis on ensuring the uniformity and miscibility of the oil phase comprising surfactant and both unmodified or iodine-modified compounds. This observation is pivotal for achieving stable and uniform nanodroplet emulsions, thereby augmenting the effectiveness and safety of the resultant contrast agents. Our spontaneous emulsification approach for producing lipid nanoemulsions saves time and money, reduces material degradation risk, and facilitates scalable production. This method ensures efficient size reduction with uniform distribution, enhancing bioavailability and shelf-life. It also enables sterile filtration with minimal clogging and transparent emulsion production, crucial for achieving stable and effective contrast agents.

Our prior efforts led to the synthesis of iodine-modified lipid compounds like cholecalciferol, α-tocopherol, and mono and triglyceride compounds as iodinated cores of successful preclinical CT imaging agents 36, 37, 45. Notably, the commercialized Fenestra™ HDVC product by MediLumine is based on our iodinated α-tocopherol LNEs, achieving a two-fold increase in iodine concentration (100 mgI/mL) compared to its previous preclinical products. This allows researchers to use lower doses for vascular and hepatic imaging in small animals.

Our concept aims to reduce excipients while maximizing iodinated molecules to enhance safety and efficiency. Our findings indicate that a surfactant-to-oil ratio (SOR) of 20-30 wt% results in enhanced stability, reduced toxicity, and improved efficiency, aligning with the clinical dose of approximately 2 mL/kg (300 mgI/kg). These formulations accumulate in the liver for about three days before clearance, making them ideal CT contrast agents. This indicates that the SOR ratio is key in varying physicochemical properties, toxicity, contrast enhancement, and clinical suitability.

Cytotoxicity assessments on RAW 264.7 macrophages and IC21 immune cells, and studies on C57BL/6 mice and porcine models, validate the safety and compatibility of our ILNEs CT agent. Histological analysis shows no necrosis or tissue damage in main organs. Comprehensive evaluations, including blood chemistry, CBC, and TSH analyses at 3 and 10 days post-injection, along with ongoing monitoring of animal behavior and weight, support the safety and potential efficacy of our ILNEs. Future investigations will focus on thorough toxicity assessments of the liver and thyroid glands across various species, facilitating the transition from preclinical research to clinical application. Our ILNEs can selectively target hepatocytes for functional and anatomical imaging, staging and monitoring fatty liver disease, and quantifying liver tumor burdens, validating their potential for heart and liver imaging.

## Conclusion

We introduce TIPhO lipid nanoemulsions (ILNEs) as a promising nano-contrast agent for clinical CT imaging, overcoming the limitations of traditional hydrophilic molecules and other nano-based materials. With a size of approximately 78 nm and minimal excipients, ILNEs are safe, scalable, and stable for intravenous use over several years. They follow the hepatic pathway and are eliminated within 72 h post-injection. *In vitro* and *in vivo* studies in small and large animal models have validated the safety and contrast enhancement of ILNEs, showing superior performance compared to preclinical (Fenestra™ HDVC) and clinical (Omnipaque™) CT agents. Our preliminary results demonstrate excellent blood pool contrast with a half-life greater than 4 h and enhanced liver imaging, with no observed pathology in the kidney, liver, or thyroid, and no adverse effects on blood function. These findings support the clinical translation of ILNEs, aiming to define the ideal nanoparticle format for safe and effective CT liver tumor imaging. We aim to assess this agent for detecting, characterizing, and monitoring treatment responses in primary and metastatic liver tumors. Ongoing non-GLP toxicology and pharmacokinetic studies in various animal species further ensure the safety of ILNEs, paving the way for their commercial use as a transformative CT contrast agent.

## Supplementary Material

Supplementary figures and tables.

## Abbreviations

CT: computed tomography
NPs: nanoparticles
ICAs: iodinated contrast agents
ILNEs: iodinated lipid nanoemulsions
TSH: thyroid-stimulating hormone
BPCA: blood pool CT contrast agent
CIN: contrast-induced nephropathy
ILNEs: iodinated lipid nanoemulsions
LDLs: low-density lipoproteins
CCK-8: cell counting kit-8
TIPhO: 2,4,6-triiodophenol oleate
SOR: surfactant-to-oil ratio
SOWR: surfactant-to-oil-to-water ratio
DLS: dynamic light scattering
NTA: nanoparticle tracking analysis
TEM: transmission electron microscopy
PTMR: particle-tracking microrheology
FBS: fetal bovine serum
HP: human plasma
HU: hounsfield units
ROI: region of interest
IC50: half-maximal inhibitory concentration
CrEL: cremophor EL
