# Supplementary material for: Toward the clinical translation of safe intravenous long circulating ILNEs contrast agent for CT imaging
Source: Theranostics. 2025 Mar 21;15(10):4550–65. doi: 10.7150/thno.110014 (PMC11984398; doi:10.7150/thno.110014)
Supplement: Supplementary file 1 — Supplementary figures and tables. [file thnov15p4550s1.pdf]

Supplementary Information

Toward the clinical translation of safe intravenous long circulating ILNEs contrast agent for CT imaging

Authors

Mohamed F. Attia[1], Ryan N. Marasco[2], Samuel Kwain[2], Charity Foxx[3], Daniel C. Whitehead[2], Alexander Kabanov[1], Yueh Z. Lee[3]**

Affiliations

[1] Center for Nanotechnology in Drug Delivery and Division of Pharmacoengineering and Molecular Pharmaceutics, Eshelman School of Pharmacy, University of North Carolina at Chapel Hill, NC 27599, USA

[2] Department of Chemistry, Clemson University, Clemson, SC, 29634, USA

[3] School of Medicine, The University of North Carolina at Chapel Hill, Chapel Hill, North Carolina 27599, USA

*Email; mattia@email.unc.edu (M. A.); and leey@med.unc.edu (Y. L.)

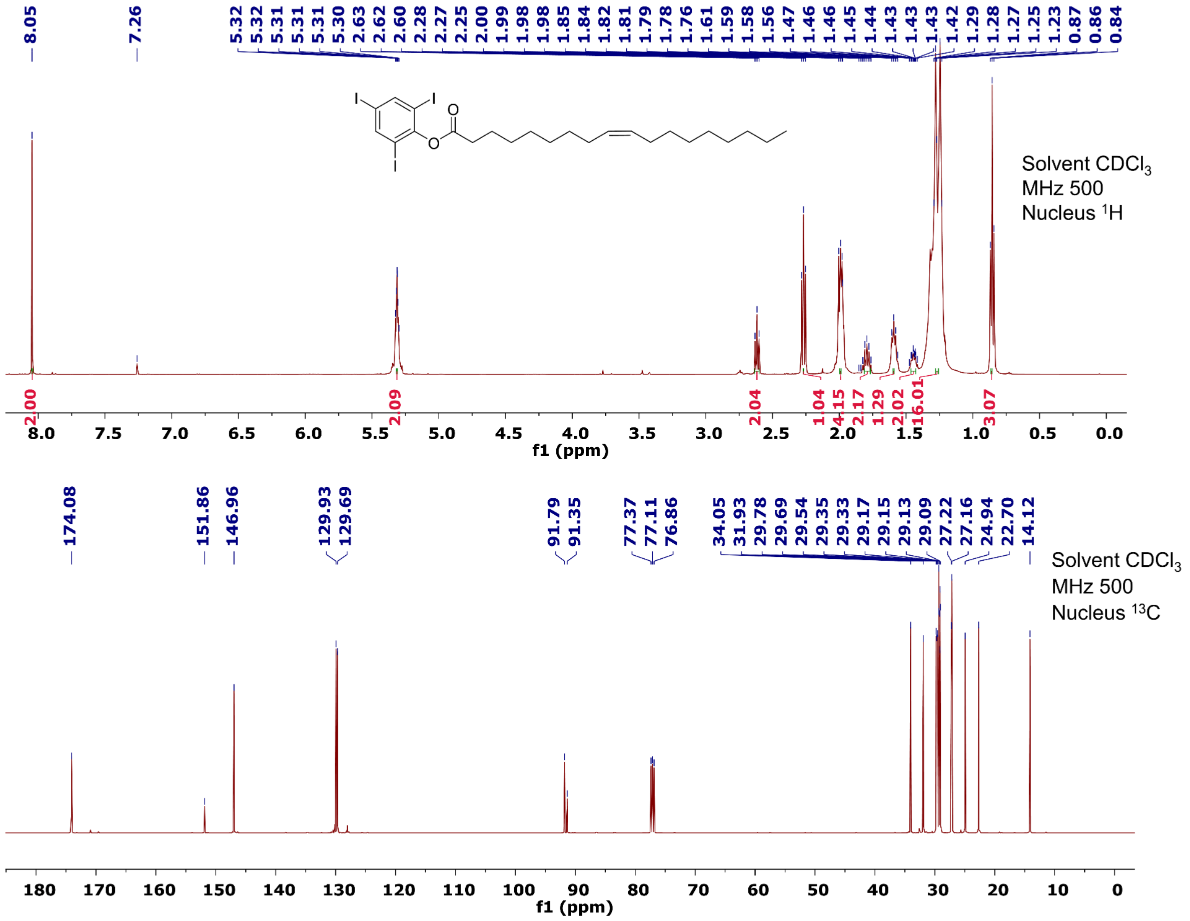

Figure S1. ^{1}HNMR (up) and ^{13}CNMR (bottom) analyses of the synthesized TIPhO.

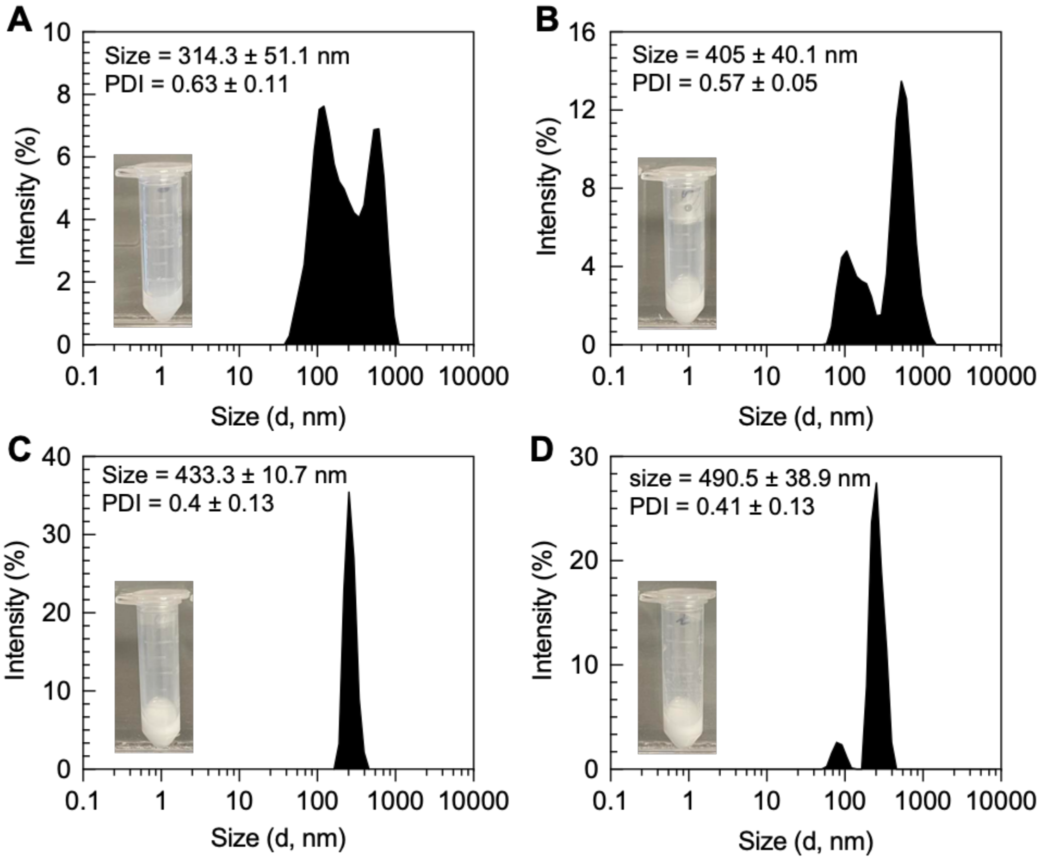

Figure S2. DLS size histograms of noniodinated LNE1–4 formulations using unmodified oleic acid as a lipid core at different SOR ratios. (a) LNE1 at SOR50, (b) LNE2 at SOR40, (c) LNE3 at SOR30, and (d) LNE4 at SOR20. Larger sizes and PDIs and possible aggregation were obtained which is typically conversely to the TIPhO LNE formulations.

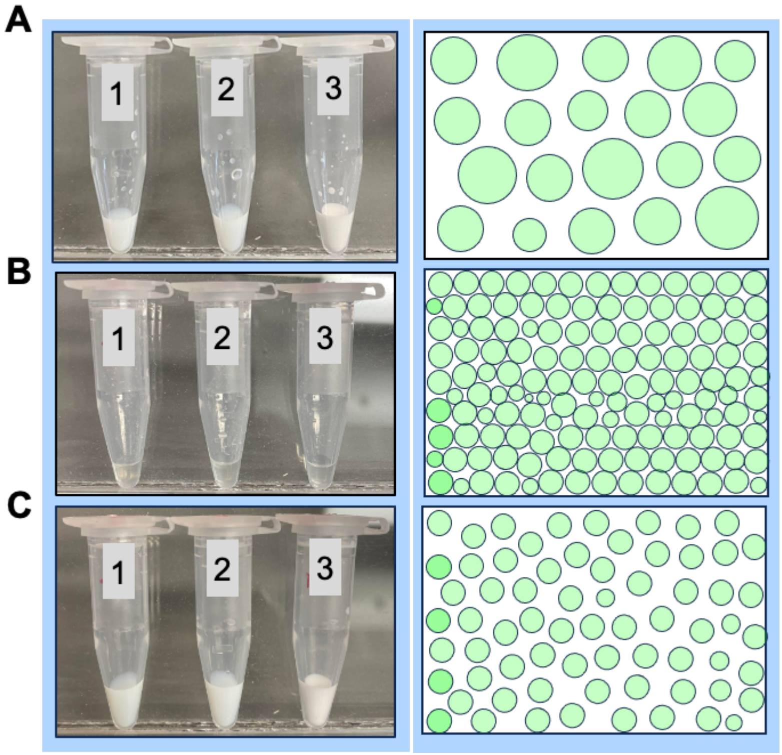

Figure S3. The appearance of ILNE formulations: (1) ILNE3 with CrEL as a surfactant at SOR30, (2) ILNE3.2 prepared with HS15 CrEL:solutol surfactants (50:50) at SOR30, and (3) ILNE4 with CrEL as a surfactant at SOR40. (A) Fresh samples after preparation immediately. (B) Lyophilized samples (oil phase). (C) reconstitution of lyophilized samples with warm 0.9% normal saline. (Right column): Schematic drawing of the nanodroplet emulsion in suspension in the three phases. The sizes and polydispersity indices (PDIs) of all samples were reduced after the freeze-drying process (see data in **Figure 2d,e** in the main manuscript).

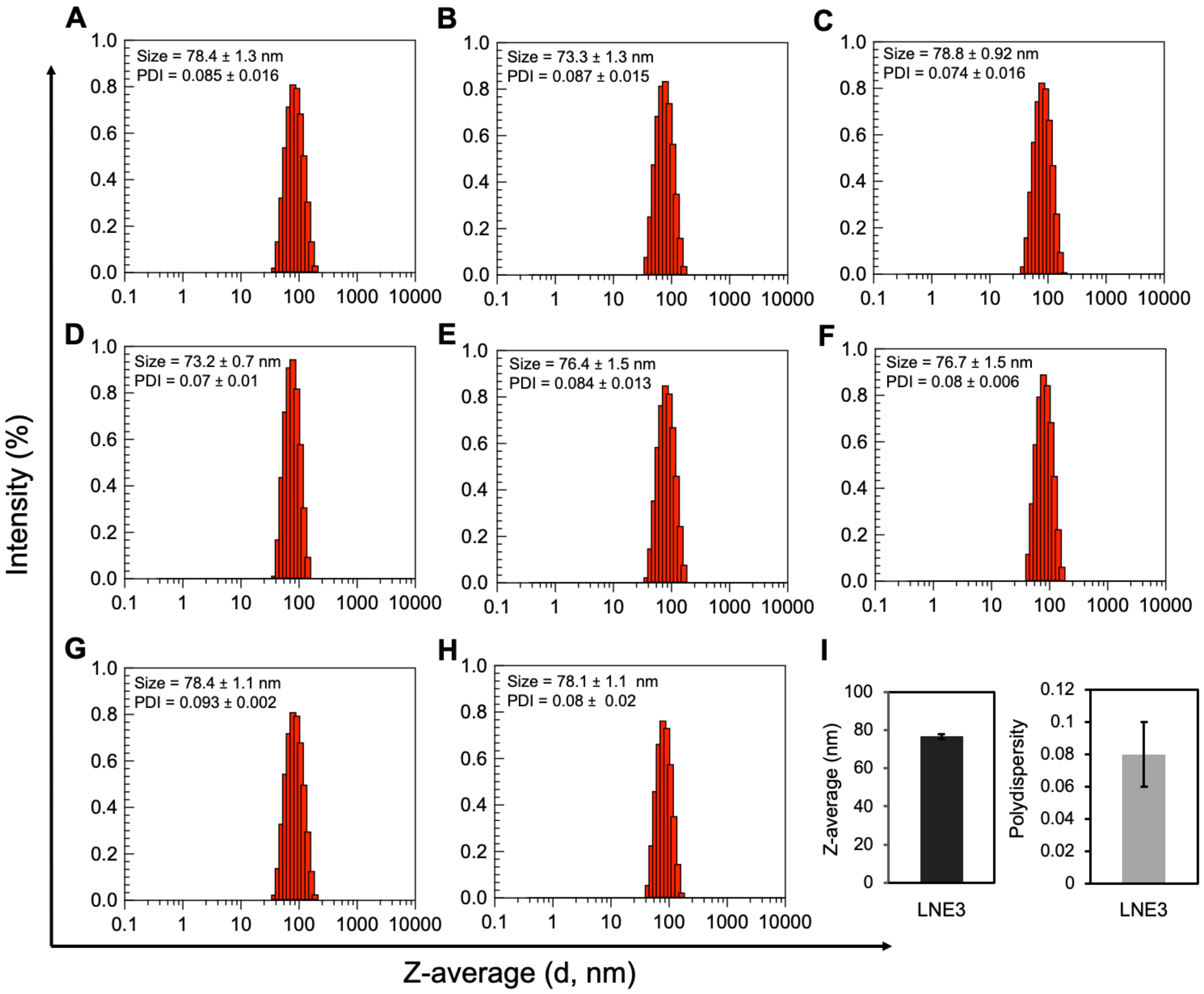

Figure S4. Batch-to-batch variation of ILNE3: (a-g) DLS histograms of different separate batches of ILNE3 formulations. (h) 35 mL batch of ILNE3. (i). The average size and polydispersity of all batches of ILNE3.

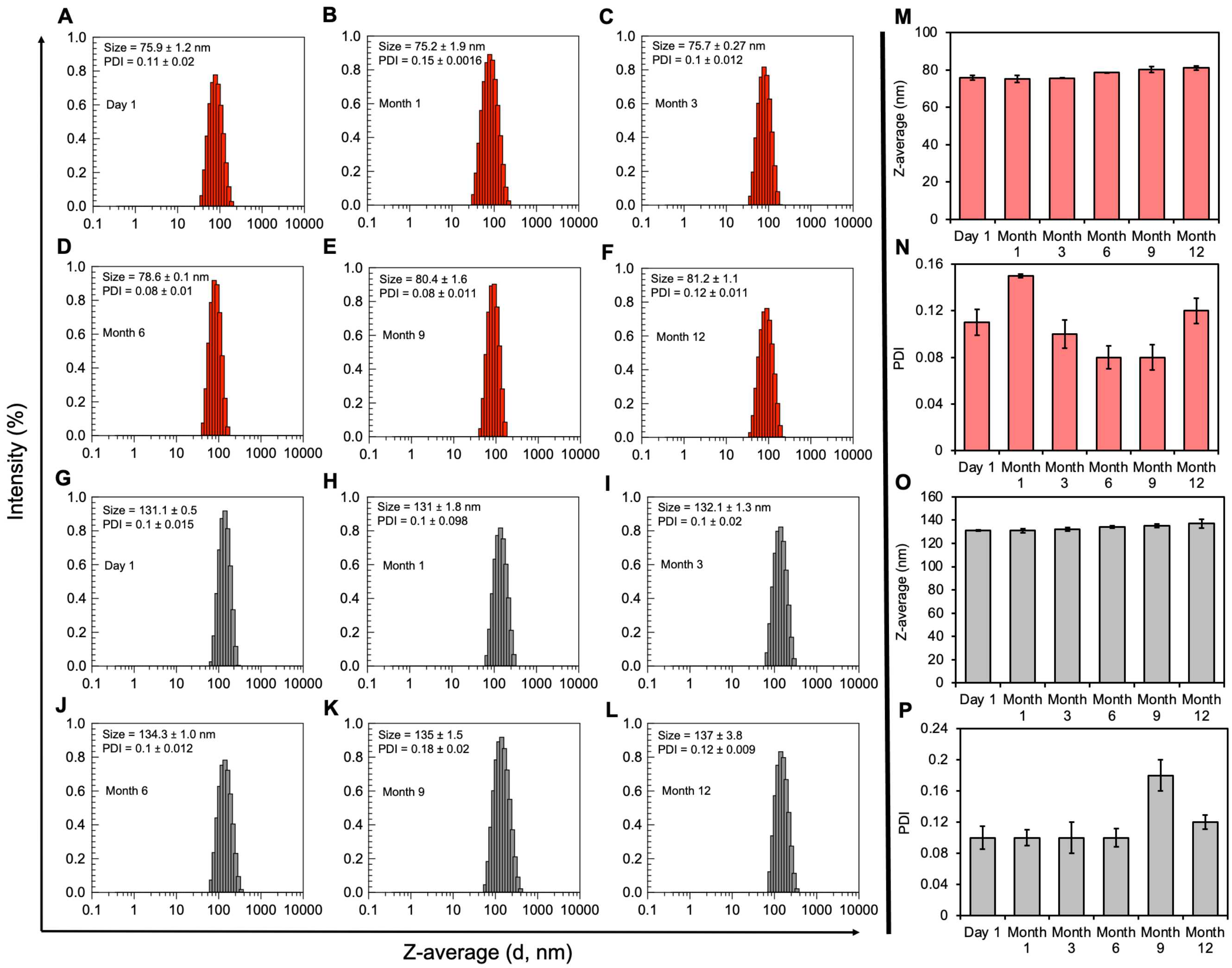

Figure S5. Shelf-life stability of both ILNE3 (a–f) and ILNE4 (g–l) formulations at room temperature over one-year post-formulation. Average size and PDI of ILNE3 (m and n, respectively). Average size and PDI of ILNE4 (o and p, respectively).

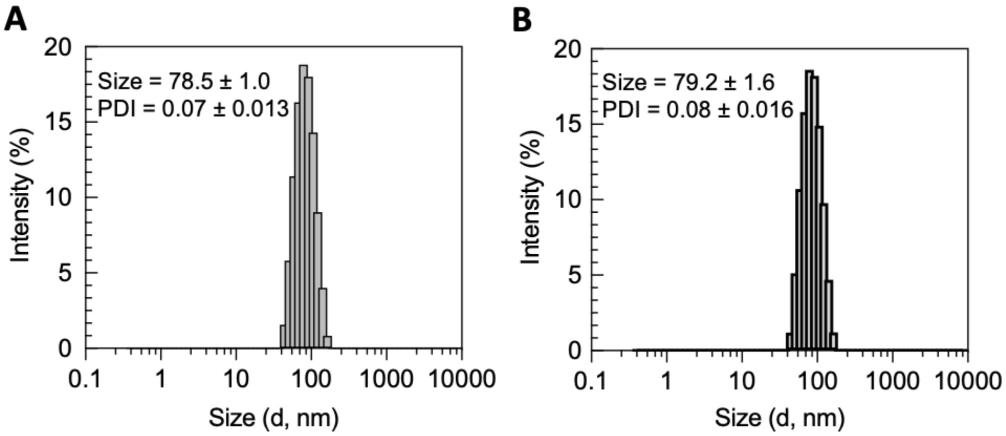

Figure S6. DLS histograms of ILNE3 stored at 4 °C. (**A**) Six months post-formulation and (**B**) nine months post-formulation.

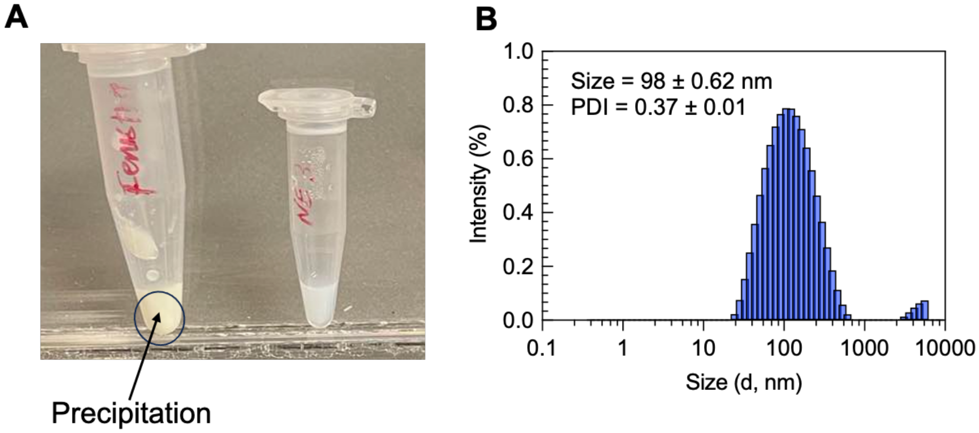

Figure S7. (a) Digital photos show the appearance of both the commercial preclinical Fenestra^{TM} HDVC with aggregates and high viscosity (left) and our ILNE3 suspension (right). (b) DLS histogram size of Fenestra HDVC with relatively high PDI.

Table S1. (a) ILNE formulations using PEGylated non-ionic surfactant Solutol® HS15 with TIPhO compound at varying SOR ratios and SOWR40. (b) ILNE formulations using mixed surfactants solutol HS15 and CrEL at a 50:50 ratio, also with TIPhO, at different SOR ratios and SOWR40.

A

LNE CT agents	SOR	Size (nm)	PDI	Iodinated compound (mg)	Surfactant (mg)	Saline (uL)	Iodine concentration (mg I/mL)		2 mL dose/kg (mgI/kg)	Surfactant (wt/v)%
							Calculated	Measured		
**LNE2.1**	40	92.1±0.96	0.22±0.01	240	160	600	119	140.6	281.2	**16**
**LNE3.1**	30	121.8±1.14	0.15±0.02	280	120	600	139	152.5	305	**12**
**LNE4.1**	20	152.3±3.3	0.2±0.01	320	80	600	159	176.5	353	**8**

B

LNE CT agents	SOR	Size (nm)	PDI	Iodinated compound (mg)	Surfactant (mg)	Saline (uL)	Iodine concentration (mg I/mL)		2 mL dose/kg (mgI/kg)	Surfactant (wt/v)%
							Calculated	Measured		
**LNE2.2**	40	256.7±25.71	0.54±0.09	240	160	600	119	140.6	281.2	**16**
**LNE3.2**	30	79.4±0.24	0.08±0.01	280	120	600	139	152.5	305	**12**
**LNE4.2**	20	155.7±0.7	0.17±0.02	320	80	600	159	176.5	353	**8**

Table S2. Injected volume doses of ILNE3 at three levels of body weight.

	**Per 20g mouse**	**Per 1kg**	**Per 70kg patient**
**ILNEs**	40 µL	2 mL	140 mL
**NP excipients**	16 mg	800 mg	56 g
**Iodine (calculated)**	5.8 mg	290 mg	20.28 g
**Iodine (found)**	6.12	306 mg	21.42 g

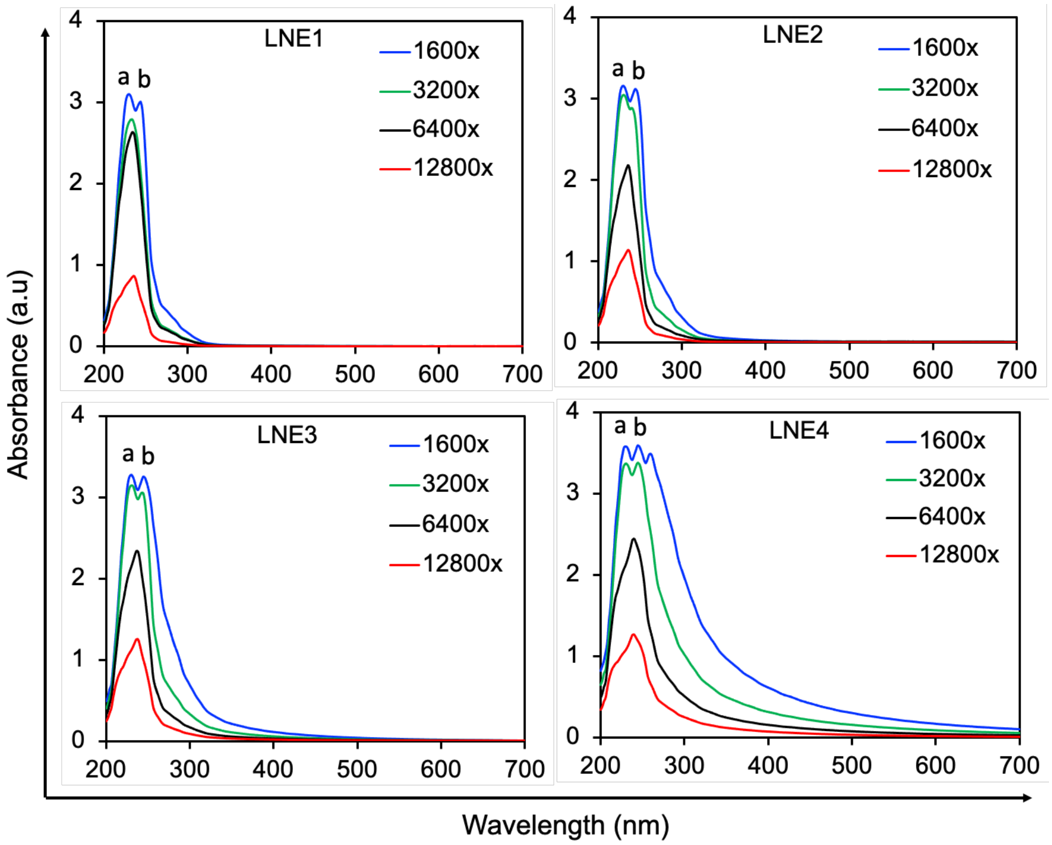

Figure S8. *UV-vis* absorption spectra of ILNE1–4 formulations at different dilutions, specifically 1600x, 3200x, 6400x, and 12800x). Two distinct peaks were observed for λ_{max} at 230 nm (a) for CrEL and 244 nm (b) for TIPhO.

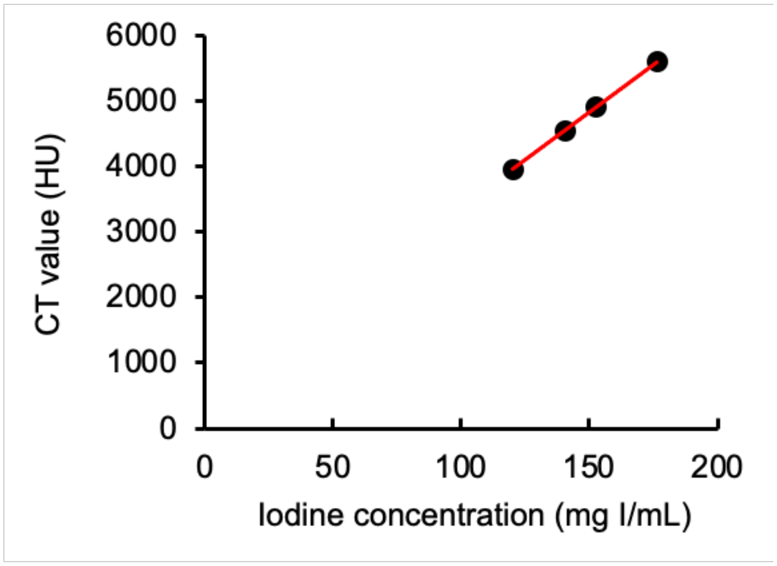

Figure S9. CT values (HU) of ILNE1–4, with a linear increase of the iodine concentration in the final suspensions.

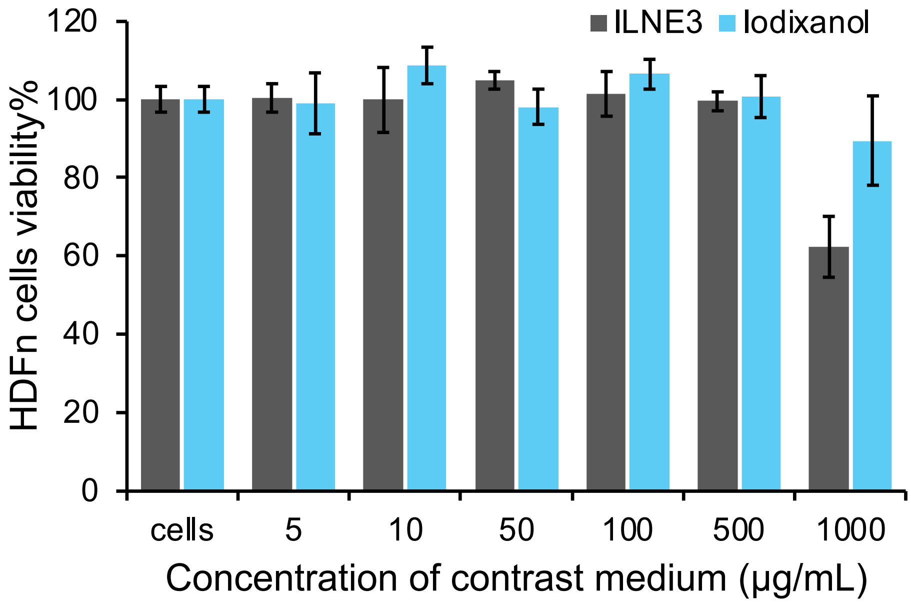

Figure S10. Cell viability assessment of human dermal fibroblasts, neonatal (HDFn) cells after treatment with ILNE3 and iodixanol for 24 hours. Data are presented as the average ± SD (n = 6).

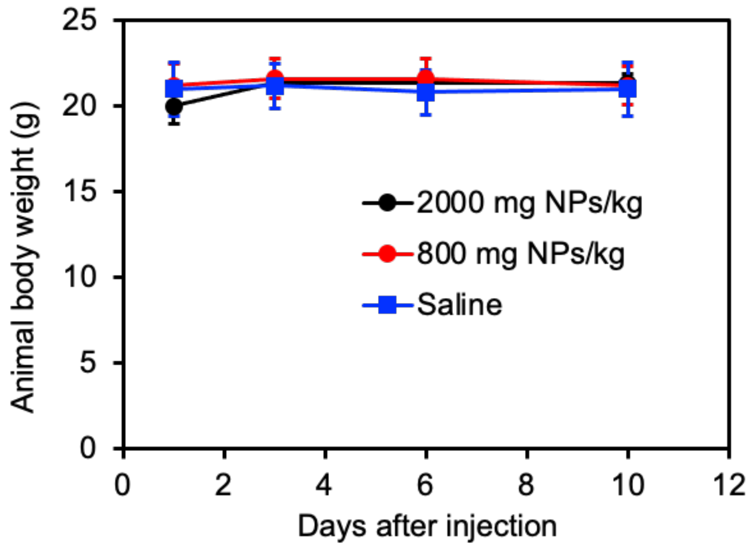

Figure S11. Animal body weight over time after injection of mice with saline (n =3), ILNE3 at a dose of 800 mg NPs/kg (n=5), and a dose of 2000 mg NPs/kg (n=3).
